# Curious Accident of the Forceps

**Published:** 1873-11

**Authors:** 


Curious Accident of the Forceps.—Dr. Wm. Goodell related the history to the Philadelphia Obstetrical Society. Hodge's Forceps were applied with great ease, to the sides of the child's head, the female blade being first introduced and rotated up under the symphysis. The lock looked directly towards the left illium. The handles, of course, pressed very firmly upon the coccyx, but the interval between them was rather greater than when the biparietal diameter is grasped. In the absence of any other manifest cause, this divergence of the handles was attributed to a large head. After an hour's hard work the head began, during traction, to bear upon the floor of the pelvis; but as the occiput now tended to rotate into the hollow of the sacrum, and, in fact, had reached the left sacroiliac synchondrosis, Dr. G. decided to end the labor with the vectis. With that view the sacral blade was first removed; but no prudent force could release the pubic one. It was firmly held by some hard body, which could just be touched by the finger, and which evidently projected through the fenestra. The sacral blade was therefore reapplied, the occiput forcibly rotated to the left acetabulum, and the head with much difficulty delivered in that oblique position. The disturbing cause was now evident enough; the *dorsal* surface of the child's right hand lay against the cheek, and the elbow, being, on this account, raised up from the body and in contact with the head, had been noosed by the fenestra of the anterior blade. The tip of the blade was deeply buried in the palmar aspect of the wrist; the proximal portion of its concave edge seemed lost in the soft tissues of the inner surface of the humerus; while all of the forearm lying between these two points projected through the fenestra. The humerus, just above its middle, was broken in two, and the epiphysis of the radius had been separated from its shaft. The child cried heartily, and otherwise, seemed none the worse for the rough usage to which it had been subjected. By the use of cardboard splints, perfect union, without any deformity, was gained in seventeen days. The mother required the catheter for four days, but made a prompt recovery in the usual time, being out of bed on the eighth day.

